# European LeukemiaNet classification intermediate risk-1 cohort is associated with poor outcomes in adults with acute myeloid leukemia undergoing allogeneic hematopoietic cell transplantation

**DOI:** 10.1038/bcj.2014.35

**Published:** 2014-05-30

**Authors:** B C Medeiros, L Tian, S Robenson, G G Laport, L J Johnston, J A Shizuru, D B Miklos, S Arai, J E Benjamin, W-K Weng, R S Negrin, R Lowsky

**Affiliations:** 1 Division of Hematology, Stanford University School of Medicine, Stanford, CA, USA; 2 Department of Health, Research and Policy, Stanford University School of Medicine, Stanford, CA, USA; 3 Division of Blood and Marrow Transplantation, Stanford University School of Medicine, Stanford, CA, USA

Acquired cytogenetic abnormalities in patients with acute myeloid leukemia (AML) are important independent predictors of response and survival.^1^ AML with normal karyotype (NK-AML) represents the largest cohort and is associated with intermediate risk. In NK-AML, distinct genomic abnormalities of the *NPM1*, *CEBPA* and *FLT3* genes provide prognostic information.^2^ The European LeukemiaNet (ELN) proposed a reporting system that included cytogenetic and molecular abnormalities.^3^ Two large studies demonstrated that the ELN classification was a powerful tool to predict outcomes in AML patients.^4, 5^ We performed a retrospective study of AML allogeneic hematopoietic cell transplantation (HCT) recipients, and determined the performance and prognostic impact of the ELN classification in this cohort.

We identified 332 consecutive adult (age ⩾18 years) AML patients who received their first allogeneic HCT following myeloablative or reduced-intensity conditioning at Stanford University between January 2000 and September 2011 (minimum follow-up: 2 years). Patients with acute promyelocytic leukemia and those who received a graft from a syngeneic, cord blood or haploidentical donor were excluded. In total, 230 allogeneic HCT from related or unrelated donors were performed in first complete remission (CR1). Of these patients, 106 patients were assigned an ELN risk group^3^ based on complete cytogenetic and molecular data extracted from source documents. Most patients (>95%) received induction chemotherapy followed by one or two cycles of consolidation therapy before transplantation. The patients underwent transplantation using a variety of myeloablative^6, 7, 8, 9^ and reduced-intensity^10, 11^ regimens. Patients received bone marrow or granulocyte colony-stimulating factor-mobilized blood from HLA-matched donors. Graft versus host disease prophylaxis consisted of a calcineurin inhibitor combined with mycophenolate mofetil or methotrexate.^10^ Baseline characteristics were reported descriptively. Continuous variables are summarized by their means and standard deviations, whereas categorical variables are summarized by proportions. Wilcoxon rank-sum test compared continuous variables and Fisher's exact test and chi-square test compared categorical variables. Overall survival (OS) was defined as the time from hematopoietic cell infusion to death from any cause. Event-free survival (EFS) was defined as the time from cell infusion to disease relapse or death from any cause, whichever occurred first. OS and EFS probabilities were estimated with the Kaplan–Meier estimators. Patients who were alive, disease free or lost to follow-up were censored at last follow-up. The log-rank test was used for comparisons of survival probabilities. A Cox regression analysis was used to adjust for potential confounders including age, intensity of conditioning regimen, time from transplant and donor relationship. All *P*-values are two-sided with a 0.05 significance.

The pretransplant characteristics of the 106 patients are shown in Supplementary Table 1. Median follow-up for living patients was 19.3 (12–144) months. Patients who received full-dose conditioning were younger and had bone marrow as the source of the graft in 22% of the cases. In patients with intermediate risk (IR)-I, nearly three-quarter (72%) of the patients received an allogeneic HCT for *FLT3-ITD+* AML. The median OS for the 106 patients was 32 months (95% confidence interval: 16.2, 47.8 months). As previously shown,^12^ conditioning intensity did not associate with survival (*P*=0.79) (data not shown).

The ELN risk classification had significant impact on OS and EFS in AML with the best outcomes noted in patients with favorable risk (FR) and IR-II, while patients with IR-I had the lowest survival (*P*=0.019) (Figures 1a and b). The hazard ratio for death was two- and threefold higher in patients with adverse risk (AR) and IR-I compared with patients with IR-II (Table 1). Cox multivariate analysis showed that AR and IR-I remained significantly associated with poorer survival (Table 1). In all, 57% (60/106) of patients died and of that, 62% (37/60) were a result of disease relapse. Multivariate Cox regression analysis demonstrated the association of the ELN classification with the cause of death. AR (*P=*0.06) and IR-I (*P*=0.05) were associated with increased relapses in these patients. The ELN classification did not predict for non-relapse mortality (Supplementary Table 2). Other cytogenetic classifications have been used to stratify the outcome of AML patients receiving an allogeneic HCT. We analyzed the outcomes from our cohort according to the Center for International Blood and Marrow Transplant Research (CIBMTR) and the Medical Research Council (MRC) classifications.^1, 12^ Unlike the ELN classification, neither cytogenetic classification stratified the outcomes for our cohort of patients (Supplementary Figures 1A–D and Supplementary Table 3).

Patients with ELN AR and IR-I had significantly decreased survival due to an increased risk of posttransplantation relapse. In keeping with our observations, the ELN classification was used to stratify young AML patients who underwent allogeneic HCT in CR1, and reported favorable outcomes for FR and IR-II patients, and short OS for AR patients.^4^ Yet unlike our findings, in their report the ELN classification did not segregate outcomes of IR-I patients from FR and IR-II patients. However, only 20% of the IR-I patients had FLT3-ITD abnormalities compared with 72% of IR-I patients in the current study. The higher percentage of FLT3-ITD abnormalities among the NK-AML patients in our study may explain these differences in outcomes. The ELN classification was a better predictor of posttransplant outcomes compared with classifications that use only cytogenetic data. When we applied the CIBMTR and MRC genomic classifications to our cohort, very few patients were FR and no significant differences were observed in the overall outcomes.

Several limitations should be considered. The study is retrospective and the population is heterogeneous, consequently unmeasured covariates might have had impact on the outcomes. Some of the ELN groups had few patients and there is variability on conditioning regimen, donor type and graft used. Finally, <50% of all patients referred for transplantation had complete cytogenetic and molecular typing performed at diagnosis. The high cumulative incidence of relapse in patients with FR was an unexpected finding. This finding likely reflects the presence of known risk factors of relapse that were not captured by the ELN classification. Retrospective review of the individual charts revealed high-risk features in this cohort, such as older age or presence of *C-KIT* and *FLT-3* mutations in 60% of FR patients.

In summary, ELN classification is an effective prognostic tool in AML patients receiving allogeneic HCT in CR1. IR-I cohort, characterized predominantly by *FLT3-ITD+* subjects, represents a poor prognostic molecular finding that adversely affected the outcomes.

## Figures and Tables

**Figure 1:**
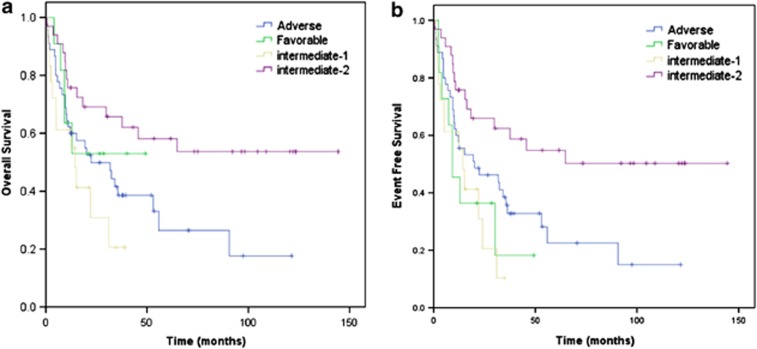
Overall and event-free survival of AML patients receiving allogeneic HCT in first complete remission. (**a**) Probability of overall survival among patients in CR1 stratified according to ELN classification. (**b**) Probability of event-free survival among patients in CR1 stratified according to ELN classification

**Table 1:** Summary of overall and event-free survival according to risk groups

*ELN category*	*%*	*Median age (range)*	*Overall survival*					*Event-free survival*				
			*OS, mos (range)*	*Unadjusted*		*Adjusted*		*Median EFS, mos (range)*	*Unadjusted*		*Adjusted*	
				*HR (95% CI)*	P*-value*	*HR (95% CI)*	P*-value*		*HR (95% CI)*	P*-value*	*HR (95% CI)*	P*-value*
Favorable	9	55 (19–72)	48% (32–64)^a^	1.52 (0.50, 4.68)	0.46	1.67 (0.53, 5.27)	0.38	9.2 (2.3–49)	2.70 (1.09, 6.69)	0.03	2.91 (1.13, 7.49)	0.027
INT-I	17	55 (19–71)	14.4 (1–39)^b^	3.18 (1.44, 7.04)	0.004	3.17 (1.38, 7.26)	0.006	22.1 (0.3–35)	3.14 (1.47, 6.71)	0.003	3.38 (1.53, 7.47)	0.003
INT-II	31	52 (17–65)	58% (49–67)^a^	1	–	1	–	66.5 (0.1–144)	1	–	1	–
Adverse	43	51 (25–73)	22.4 (0.2–121)^b^	2.19 (1.16, 4.13)	0.01	2.23 (1.11, 4.48)	0.025	35.6 (0.2–121)	2.21 (1.2, 4.05)	0.01	2.53 (1.30, 4.92)	0.006

Abbreviations: CI, confidence interval; ELN, European LeukemiaNet; HR, hazard ratio; INT, intermediate; Mos, months.

Summary of allogeneic transplant outcomes according to ELN classification, restricted to AML patients in CR1. Cox regression analyses used to determine the unadjusted and adjusted hazard ratio for OS and EFS.

a 48-Month survival for the cohort.

b Median overall survival.
