# Supplementary material for: European LeukemiaNet classification intermediate risk-1 cohort is associated with poor outcomes in adults with acute myeloid leukemia undergoing allogeneic hematopoietic cell transplantation
Source: Blood Cancer J. 2014 May 30;4(5):e216–. doi: 10.1038/bcj.2014.35 (PMC4042305; doi:10.1038/bcj.2014.35)
Supplement: Supplementary Information [file bcj201435x1.doc]

**Supplemental Figure 1 - Overall and event-free survival according to cytogenetic classifications not incorporating molecular information**

A- Probability of overall survival and B- Probability of event-free survival among patients in our cohort stratified according to the CIBMTR classification.

C- Probability of overall survival and D- Probability of event-free survival among patients in our cohort stratified according to MRC classification.


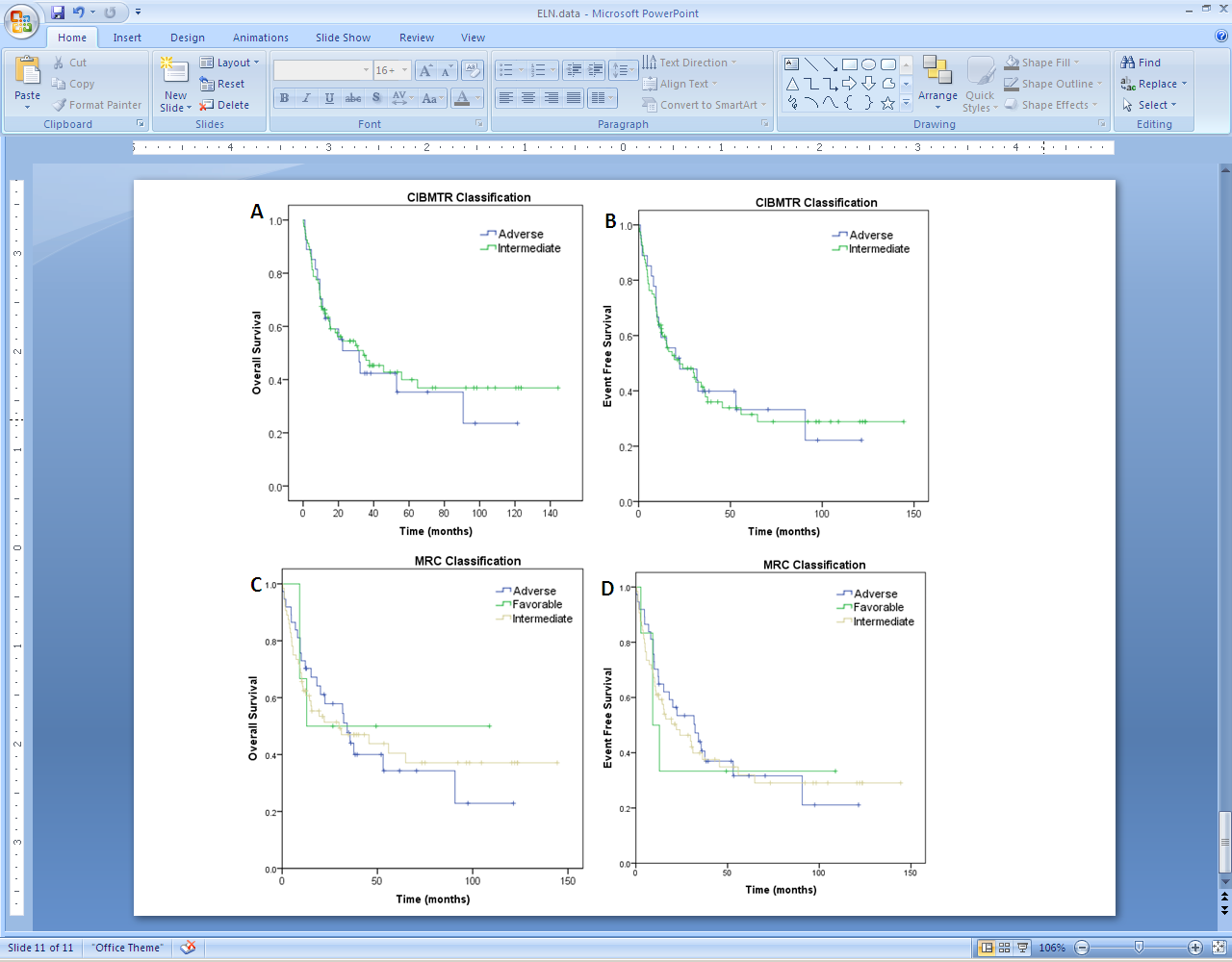


**Supplemental t**able 1. Baseline patient characteristics according to conditioning regimen

|  | **Total no. (%)** | | |  |
| --- | --- | --- | --- | --- |
|  | **Total (n -106)** | **MAC (n-59)** | **RIC (n-47)** | ***p-value*** |
| **Median age, y (range)** | 52 (18-73) | 45 (18-61) | 58 (46-73) | <0.001^1^ |
| **ELN Classification (%)** |  |  |  | 0.23^2^ |
| Favorable | 10 (9) | 5 (9) | 5 (11) |  |
| Intermediate-1 | 18 (17) | 9 (15) | 9 (19) |  |
| Intermediate-2 | 33 (31) | 15 (25) | 18 (38) |  |
| Adverse | 45 (42) | 30 (51) | 15 (32) |  |
| **Female (%)** | 53 (52) | 31 (53) | 25 (53) | 0.15^3^ |
| **Donor match (%)** |  |  |  | 0.12^3^ |
| MRD | 61 (57) | 36 (61) | 25 (53) |  |
| MUD | 45 (43) | 23 (39) | 22 (47) |  |
| **Graft Source (%)** |  |  |  | <0.01^3^ |
| BM | 12 (11) | 12 (20) | 0 (0) |  |
| PB | 94 (89) | 47 (80) | 47 (100) |  |

**Abbreviations:** ELN – European LeukemiaNet; MAC – Full dose myeloablative conditioning; RIC - Reduced Intensity Conditioning; HSCT – Hematopoietic stem cell transplant; CR – Complete remission; MRD – Matched related donor; MUD – Matched unrelated donor; BM – Bone marrow; PB – Peripheral blood

Statistical Calculations used:

1- Wilcoxon rank-sum test, 2- Chi-Square Test; 3- Fisher's exact test

**Supplemental table 2. Proportional Cox regression model for relapse and non-relapse mortality for AML patients in CR1 according to risk groups**

| **Relapse-specific mortality** | | | |
| --- | --- | --- | --- |
|  | HR | 95% CI | *p*-value |
| Favorable | 1.51 | [0.39, 5.81] | 0.5 |
| INT-I | 2.80 | [1.00, 7.82] | 0.05 |
| INT-II | 1 | -- | -- |
| Adverse | 2.30 | [0.98, 5.42] | 0.06 |
| **Non-relapse mortality** | | | |
|  | HR | 95% CI | *p*-value |
| Favorable | 1.76 | [0.19, 16.6] | 0.6 |
| INT-I | 3.34 | [0.80, 14.0] | 0.01 |
| INT-II | 1 | -- | -- |
| Adverse | 1.92 | [0.56, 6.53] | 0.3 |

**Abbreviations:** HR – Hazard ratio; INT-I – Intermediate-1; INT-II- Intermediate-2.

**Supplemental table 3 – Outcomes according to the CIBMTR and MRC cytogenetic classification**

|  | **Overall Survival** | | | | **Event Free Survival** | | | |
| --- | --- | --- | --- | --- | --- | --- | --- | --- |
|  | **Unadjusted** | | **Adjusted** | | **Unadjusted** | | **Adjusted** | |
|  | **HR**  **(95% CI)** | ***p-value*** | **HR**  **(95% CI)** | ***p-value*** | **HR**  **(95% CI)** | ***p-value*** | **HR**  **(95% CI)** | ***p-value*** |
| **CIBMTR** | | | | | | | | |
|  | 0.89  (0.51, 1.57) | 0.69 | 0.96  (0.54, 1.73) | 0.9 | 0.93  (0.54, 1.59) | 0.79 | 0.92  (0.52, 1.62) | 0.76 |
| **MRC** | | | | | | | | |
|  | 0.83  (0.25, 2.7) | 0.1 | 0.93  (0.53, 1.63) | 0.53 | 0.99  (0.53, 1.83) | 0.14 | 1.21  (0.62, 2.32) | 0.85 |

**Abbreviations:** HR – Hazard ratio; CIBMTR – **Center for International Blood and Marrow Transplant Research**; MRC – Medical Research Council, CI- Confidence Interval
